# Diagnostic performance of respirators for collection and detection of SARS-CoV-2

**DOI:** 10.1038/s41598-023-39789-w

**Published:** 2023-08-15

**Authors:** Hwang-soo Kim, Hansol Lee, Seonghui Kang, Woo Joo Kim, Sehyun Shin

**Affiliations:** 1 Department of Micro-nano System Engineering, Korea University, Seoul, 02841 Republic of Korea; 2 Asia Pacific Influenza Institute, Korea University College of Medicine, Seoul, 08308 Republic of Korea; 3 Division of Infectious Diseases, Department of Internal Medicine, Konyang University Hospital, Daejeon, 35365 Republic of Korea; 4 Division of Infectious Diseases, Department of Internal Medicine, Korea University College of Medicine, Seoul, 08308 Republic of Korea; 5 School of Mechanical Engineering, Korea University, Seoul, 02841 Republic of Korea

**Keywords:** Biological techniques, Health care, Pathogenesis

## Abstract

Respirators, called as face mask, have been used to protect the wearer from the outside harmful air environment and prevent any virus from being released to neighbors from potentially infected exhaled breath. The antiviral effectiveness of respirators has not only been researched scientifically, but has also become a global issue due to society's obligation to wear respirators. In this paper, we report the results of a study on the collection and detection of viruses contained in exhaled breath using respirators. The inner electrostatic filter was carefully selected for virus collection because it does not come in direct contact with either human skin or the external environment. In the study of a healthy control group, it was confirmed that a large amount of DNA and biomolecules such as exosomes were collected from the respirator exposed to exhalation, and the amount of collection increased in proportion to the wearing time. We conducted experiments using a total of 72 paired samples with nasopharyngeal swabs and respirator samples. Out of these samples, fifty tested positive for SARS-CoV-2 and twenty-two tested negative. The PCR results of the NPS and respirator samples showed a high level of agreement, with a positive percent agreement of ≥ 90% and a negative percent agreement of ≥ 99%. Furthermore, there was a notable level of concordance between RCA-flow tests and PCR when examining the respirator samples. These results suggest that this is a non-invasive, quick and easy method of collecting samples from subjects using a respirator, which can significantly reduce the hassle of waiting at airports or public places and concerns about cross-contamination. Furthermore, we expect miniaturized technologies to integrate PCR detection into respirators in the near future.

## Introduction

The infection of the severe acute respiratory syndrome coronavirus 2 (SARS-CoV-2) spread worldwide faster than any other viruses, which is considered to be a characteristic of the respiratory virus and the development of transportation. Immediately after the outbreak, many countries made respirator wearing mandatory, preventing the spread of the infection to some extent. As scientific papers report that a respirator is effective in blocking the spread of the virus^1,2^, wearing a mask has become a basic global etiquette in the pandemic scenario. This phenomenon has completely changed the concept of traditional mask wearing. In other words, the conventional purpose of wearing a mask was to protect the wearer from harmful external air. However, the purpose of the recent mandatory wearing of a mask during pandemic is to prevent the spread of the virus to people around the wearer from the air exhaled by the wearer, which can be a potential source of infection^3^. This is commonly referred as source control.

The distinction between facemasks and respirators is worth noting, as the term 'face mask' has been commonly used by the general public without proper differentiation. This has led to confusion in defining respiratory infection control measures, such as distinguishing between respirators and facemasks. To clarify, a facemask is intended for use as a source control measure by the general public and healthcare personnel, as per regulations or recommendations during the COVID-19 pandemic^4^. It is important to note that facemasks may or may not meet specific fluid barrier or filtration efficiency standards. Therefore, they cannot serve as substitutes for respirators or surgical masks. On the other hand, a respirator is a respiratory protective device designed to minimize the wearer's exposure to external airborne contaminants, including viruses present in exhaled breath^5^. Respirators, such as N95 or KF94 masks, must be certified, carefully selected, and used as part of a comprehensive respiratory protection program. It is essential to acknowledge that N95 and KF94 Filtering Facepiece Respirators (FFRs) are a subset of respirators. Lastly, surgical masks are fluid-resistant and serve as physical barriers to protect users from hazards like splashes of large droplets of blood or body fluids^4^. These masks, labeled as surgical, isolation, dental, or medical procedure masks, are regulated under the FDA certification program. Throughout this article, we use the term 'respirator' to refer specifically to N95 or KF94-certified FFR.

Note that N95 and KF94-certified respirators consist of three or four membranes, utilizing mechanical and electrostatic filtration in the middle membrane^6^. One of the interlayer membranes uses permanently charged electret fibers as a filter medium and can collect 95% of aerosolized viruses^7^. Due to the nature of certified respirators, viruses expelled through exhaled breath can be efficiently collected in respirators^8^. Recent studies have reported that SARS-CoV-2 viral RNA was collected from respirators and quantified using reverse transcription polymerase chain reaction (RT-PCR)^9–11^. Furthermore, facial respirators were used with wearable sensors for the direct detection of viruses collected from a wearer’s exhaled breath^12,13^. We also reported preliminary data for the collection of viruses using a respirator and demonstrated the feasibility to detect viruses with either RT-PCR or rolling circle amplification (RCA) methods^14^.

A few studies have reported that exhaled breath can be used as an alternative sample instead of nasopharyngeal swabs (NPSs) or saliva^15,16^. The breath test has long attracted interest for potential applications in medical diagnosis and disease monitoring^17^. This interest is primarily owing to its characteristics of non-invasiveness and easy sampling, as well as repetitive sampling from human breath. Thus, breath analysis via an electronic nose is a technique oriented around volatile organic compound (VOC) profiling in exhaled breath for diagnostic and prognostic purposes^18^. This approach, when supported by methodologies for VOC identification, has been often referred to as metabolomics or breathomics. Furthermore, efforts to detect COVID-19 with an electronic nose have been conducted^19^. However, progress from laboratory setting to routine clinical practice has been slow because of various problems including unstandardized sampling methods and the different sensitivities of adopted sensors. Moreover, the diagnostic performance of electronic noses without molecular amplification has been inferior to that of conventional PCR. Since testing of infectious viruses such as COVID-19 does not allow any false results greater than 0.1% for sensitivity, the electronic nose cannot be used in clinical practice.

**Figure 1:**
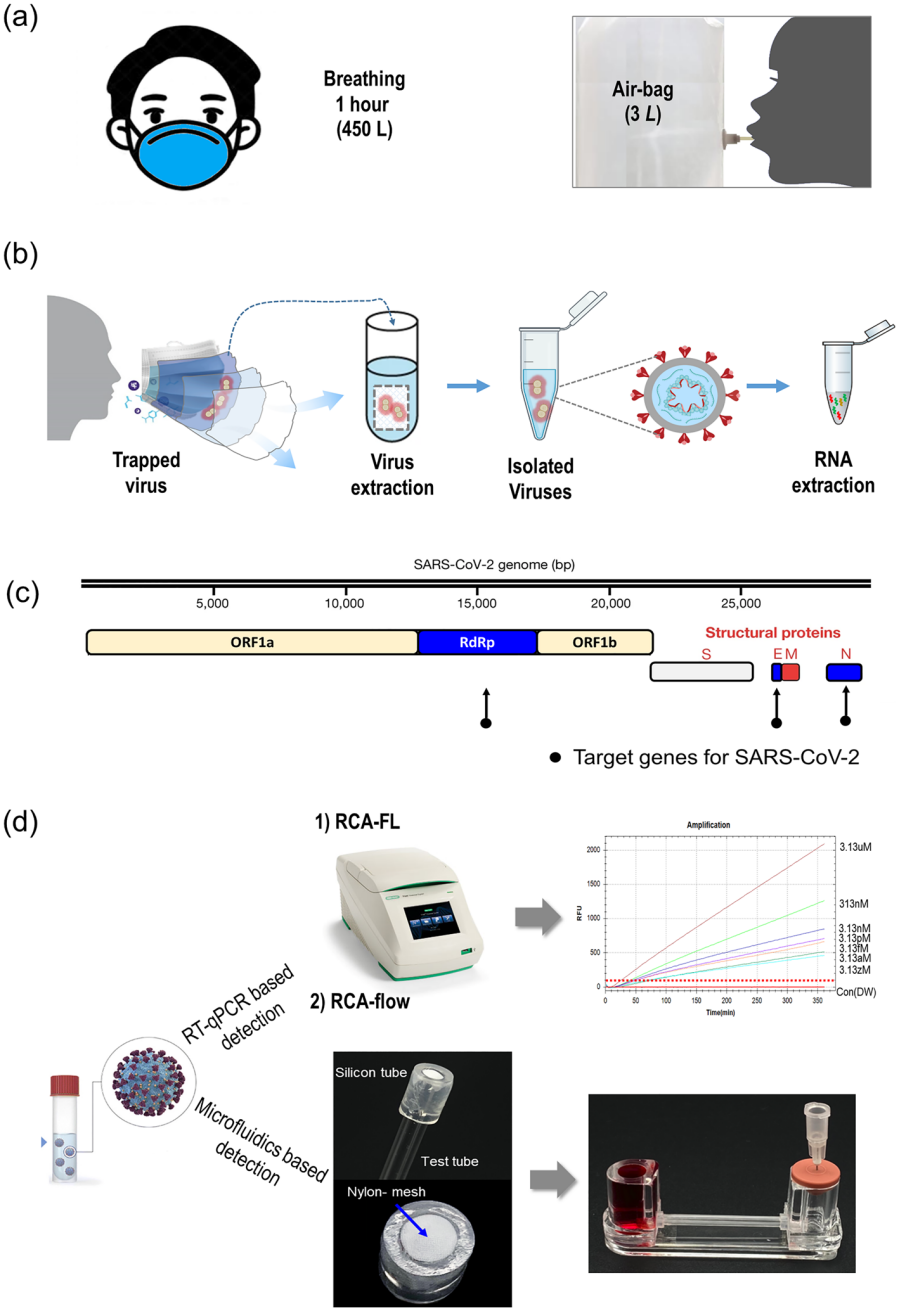
(**a**) Virus collection from exhalation to mask. (**b**) Schematic of the extraction of viral RNA from the mask. (**c**) Adoption of three target genes: N, E, and RdRp to identify SARS-CoV-2. (**d**) Adoption of two tests based on RCA. (1) General RCA test (RCA-FL) involving fluorescence detection using a PCR device, and (2) rapid test (RCA-Flow) using DNA hydrogel formation in microfluidic pores.

In a recent preliminary study, we investigated the use of exhaled breath biopsy via respirators as a potential alternative method for virus collection^20^. Our findings demonstrated that the PCR test based on respirator samples exhibited comparable performance to the PCR test using nasopharyngeal swab samples. These results suggest that exhaled breath biopsy via respirators could be a promising approach for virus detection and monitoring. Therefore, we conducted a full clinical study of collecting respirators from COVID-19 infection confirmed patients as well as healthy controls. Processed samples were examined with either a certified RT-PCR compared with authentic sampling such as NPSs, as shown in Fig. 1. In addition, these PCR results with respirator samples were compared with the RCA method that was developed in our previous study^20,21^. If successful, this methodology can provide a rapid, non-invasive sampling protocol at points-of-care and air travel checkpoints.

## Results

### Collection of SAR-CoV-2 from respirators

**Figure 2:**
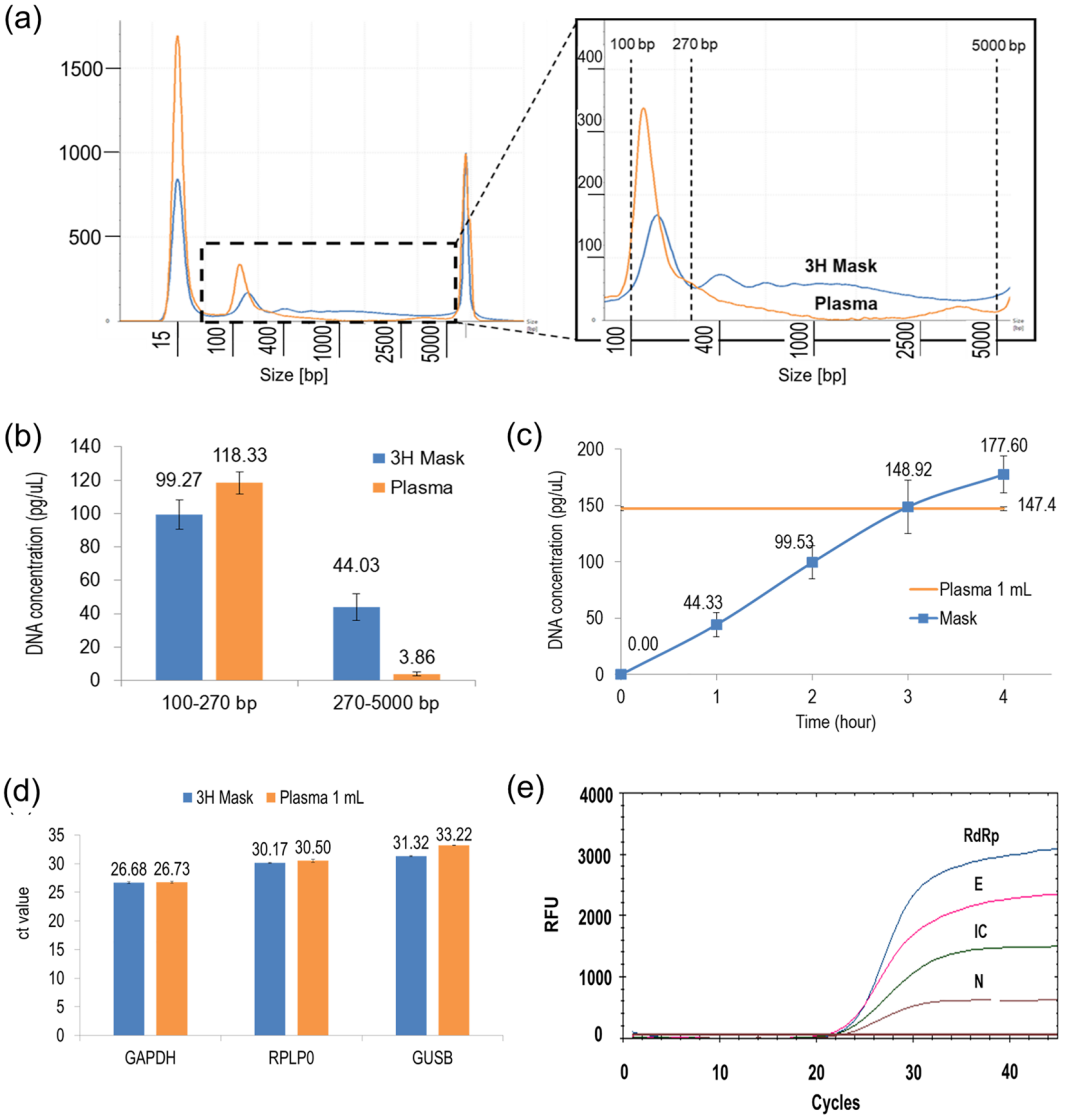
(**a**) Comparison of nucleic acid profiles of nucleic acids extracted from masks. (**b**) Quantitative comparison of the distribution of nucleic acids (nucleic acids are primarily distributed in the vicinity of 100–270 bp). (**c**) Measurement (quantity) of collected nucleic acid concentration according to mask wearing time. (**d**) Comparison of Ct values of nucleic acids for the three housekeeping genes GAPDH, GUSB, and RPLP0 in human plasma and mask. (**e**) Typical PCR results from a clinical sample of a mask worn by a patient with SAR-CoV-2 infection.

The comparison result of the nucleic acid profile of cell-free DNA (cfDNA) extracted from human plasma and nucleic acid extracted from a respirator worn for 3 h is shown in Fig. 2a. The cfDNA was distributed between 100 and 300 bp, the respirator nucleic acid was distributed between approximately 200 and 300 bp. Additionally, when quantitatively comparing the distribution of these nucleic acids, as shown in Fig. 2b, the nucleic acids were primarily distributed between 100 and 270 bp, and the concentration of the respirator nucleic acid was close that of cfDNA extracted from plasma. Therefore, the method of extracting nucleic acids from the respirator was established to some extent. As shown in Fig. 2c, the amount of extracted RNA increased almost linearly with wearing time, and the amount of DNA extracted from respirators worn for more than 3 h was even greater than that of plasma. Therefore, we recommend collecting respirators worn by patients for more than 3 h.

In this study, RT-qPCR for three housekeeping genes (GAPDH, GUSB, and RPLP0) was used and compared with human plasma to confirm the extraction performance and applicability of nucleic acids isolated from respirators. As shown in Fig. 2d, for each housekeeping gene, the Ct value of the nucleic acid isolated from the respirator was measured at a level almost similar to that of plasma; thus, we could again confirm the extraction performance of the nucleic acid from the respirator. In addition, we confirmed that a sufficient amount of nucleic acid was extracted to be used for molecular diagnosis. Figure 2e shows typical PCR results in which clinical samples were collected from respirators worn by SAR-CoV-2 infected patients. The internal control signal was first observed at cycle 24.3, while the signals for the RdRp, N and E genes were observed at cycles 34.6, 37.1 and 37.6, respectively. The current results for the respirator are identical to the PCR results for the NPS sample. In conclusion, we confirm that virus collection using respirators is possible and can be used for diagnosis.

### Nucleic acid collection performance of respirators

**Figure 3:**
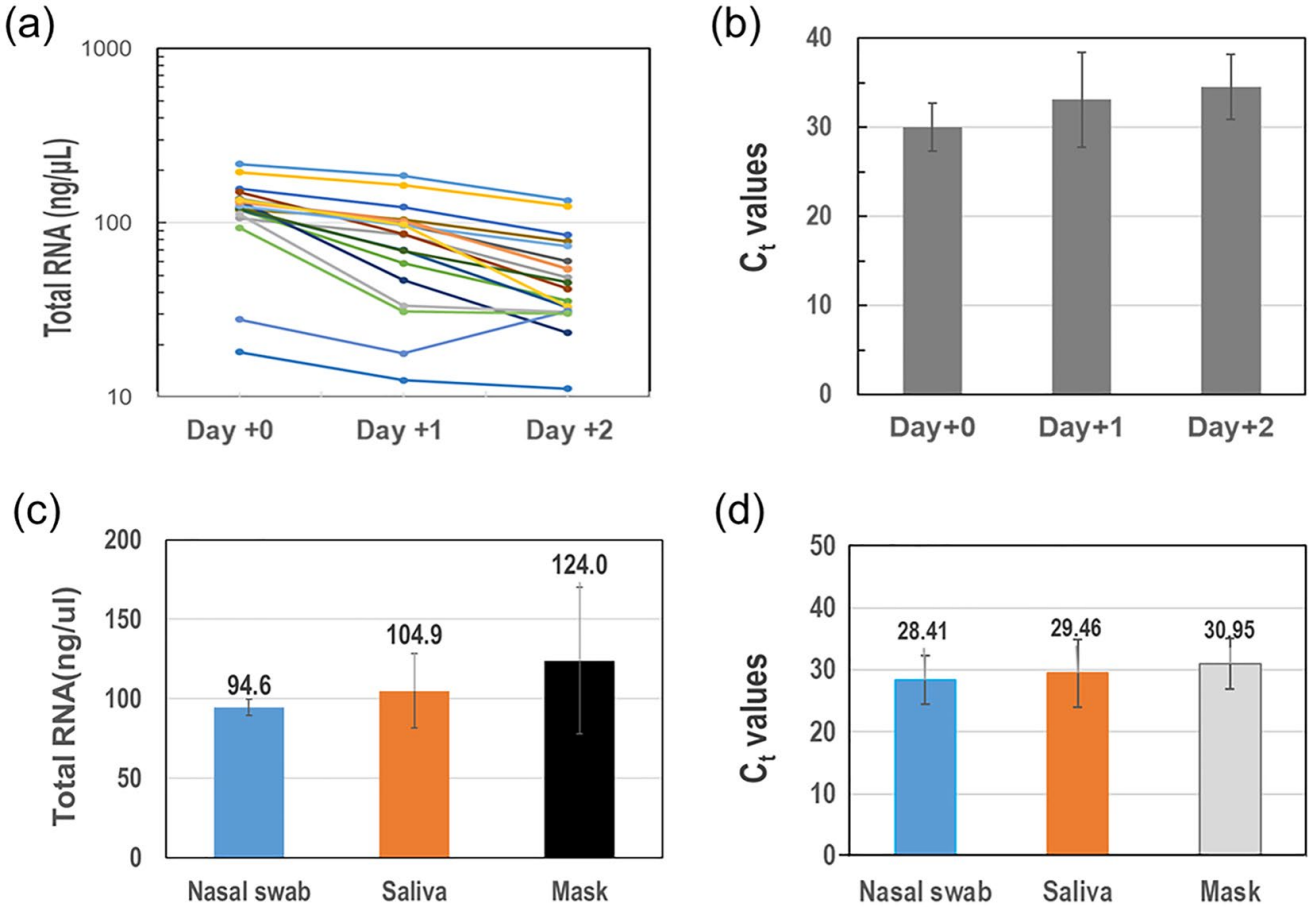
(**a**) Trend line of the amount of nucleic acids in clinical samples extracted from masks according to each collection day (n = 19). (**b**) Comparison of Ct values for target genes using clinical samples extracted from masks on each collection day. (**c**) Comparison of the amount of nucleic acids extracted from throat swabs, saliva, and masks. (**d**) Comparison of Ct values for target genes using clinical samples extracted from different samples.

As shown in Fig. 3a, the amount of nucleic acid decreased over time compared with the amount of nucleic acid on the day of collection. Additionally, PCR tests were performed on the target gene using clinical samples extracted from the respirator on each collection day to confirm each Ct value. As shown in Fig. 3b, the Ct value increased. As in the quantitative results, the amount of nucleic acids was decreasing. Therefore, we observed that the amount of nucleic acid and detection performance decreased as the collection date elapsed, and we inferred that the sensitivity can be improved if the sampling schedule is as early as possible from the date of confirmed virus infection.

The amounts of nucleic acids extracted from throat swabs, saliva, and respirators were respectively compared. As shown in Fig. 3c, the amounts were 94.6 ng/µL in the throat swab, 104.9 ng/µL in the saliva, and 124.0 ng/µL in the respirator. The sample used for this was the sample on the day of collection and N = 3. Figure 3d shows the Ct value obtained by performing PCR on the target gene using the clinical specimen extracted from each sample. As a result, the respective Ct values were 28.41, 29.46, and 30.95, confirming that there was no significant difference depending on the type of sample. Overall, we can predict that the nucleic acid collection performance from the respirator is not significantly different from the conventional method used for sample collection.

### Diagnostic performance of respirators with RT-PCR

As shown in Table 1, we conducted experiments using a total of 72 paired samples (NPS and Respirator), and the results are presented in Table 1. Among the 72 samples, 50 positive samples were collected from patients who tested positive for SARS-CoV-2 using a PCR test prior to their hospital admission. In contrast, 22 negative samples were collected from patients who were already admitted to the hospital for reasons unrelated to COVID-19 and confirmed negative through the PCR test. From the 72 hospitalized patients, we collected two types of samples, namely NPS and respirator samples, on the first day after hospitalization. As shown in Table 1, NPS sampling combined with RT-PCR test, which was adopted as a reference method to compare the diagnostic performance of respirators, reconfirmed 50 out of the 50 true positives and 22 out of 22 true negatives. Regarding the respirator sample tests, out of the 50 true positive samples, 45 were identified as positive, and all 22 true negative samples were correctly identified as negatives. Consequently, the corresponding sensitivity and specificity were calculated as 90% and 100%, respectively.

**Table 1:** Comparative analysis of PCR test results for two sampling methods (nasopharyngeal swab and respirator).

PCR test		Nasopharyngeal swab samples (reference method)	
		Positive (total 50)	Negative (total 22)
Respirator samples (Mask samples)	Positive	45	0
	Negative	5	22
Sensitivity/specificity		90% (45/50)	100% (22/22)

### Diagnostic evaluation of RCA-flow test for respirator samples

Respirator samples were evaluated with RT-PCR test and RCA-flow, as listed in Table 2. It is worth of note that the samples used in Table 2 were a subset of the samples used in Table 1. TH results of RT-PCR were used as reference to evaluate the diagnostic performance of RCA-flow test. Among 26 positive results of RT-PCR, 24 were positive and 2 were negative in RCA-flow test, whereas All of 22 negatives in RT-PCR were negative in RCA-flow test. Therefore, the sensitivity and specificity were numerically 92.3% and 100%, respectively. In the literature evaluating the comparative analysis and clinical performance of the Allplex 2019-nCoV real-time PCR (Seegene, Seoul, Korea) kit and several RT-PCR kits, the detection sensitivity of positive samples ranged 92.8–95.9%^22^. These results indicate that the performance of RCA-flow is not significantly different from the diagnostic results of RT-PCR, considering that RCA-flow is a point-of-care-testing-based diagnostic technique.

**Table 2:** Comparison of sensitivity and specificity according to test methods using clinical samples.

Respirator samples		RT-PCR test (Seegene™)	
		Positive	Negative
RCA-flow	Positive	24	0
	Negative	2	22
Sum		26	22
Sensitivity/specificity		92.3% (24/26)	100% (22/22)

## Discussion

In this study, we aimed to explore the feasibility of using respirators as a virus collection tool to simplify the current complex and inconvenient sample collection protocol. The sensitivity of SARS-CoV-2 detection using samples extracted from respirators was found to be 90% of that of nasopharyngeal swab samples used as the benchmark in this study. These findings differ from those reported in a recent study^23^, which could be attributed to differences in respirator-wearing time and sample size. For example, their respirator-wearing time was limited to 30–60 min, whereas our study required a minimum of 3 h. As illustrated in Fig. 2c, the amount of extracted RNA increased almost linearly with wearing time. Additionally, discrepancies between the two studies may have arisen due to differences in sample collection time from the date of COVID-19 detection^24^. As shown in Fig. 3a, the amount of RNA extracted from the respirator sample exponentially decreases with time. With the passage of time, PCR Ct values keep increasing and may exceed the diagnostic cutoff value (Fig. 3b).

An important feature of respirators is that they are exposed to a large volume of breathed air for prolonged periods. The amount of exhaled breath supplied to a respirator per hour varies among individuals, but on average, it is approximately 450 L when at rest. Therefore, if collected for about 3 h, it amounts to 1350 L. In contrast, the maximum air volume that can be collected at once using devices ranges from 0.2 to 3 L, which might result in low sensitivity to identify infected patients^23^. Furthermore, respirators can capture a large amount of viruses, and this viral load continues to increase for up to 4 h (Fig. 2b).

This study demonstrated that respiratory viruses from infected people can be effectively collected from certified respirators such as KF94 or N95. The results of this study suggest how important personal protective equipment (PPE) is for preventing the spread of infectious viruses and for personal isolation. Although many studies have reported that respirators can block respiratory aerosols^2,8^, this study confirmed the collection of respiratory viruses in respirators. In other words, the respiratory virus that is continuously released to the outside of the infected person through breathing can be effectively confined with the respirator. However, a potential limitation of this study is the risk of mask contamination by participants or external sources during the donning and doffing process, which may include inadvertent contact with contaminated hands. The risk of such contamination increases with prolonged mask usage (beyond 6 h) and higher rates of clinical contact^25^. To mitigate this risk, protocols regarding the duration of mask use should specify a maximum time for continuous use and consider guidance tailored to high contact settings.

In this study, however, several issues in respirator sampling were identified and the development of a new method to efficiently extract viral RNA from different respirators needs to be addressed. Specifically, the respirator samples were compared with other biological fluids such as saliva, mucus, and sputum, including NPS, to compare the capture and extraction ability of nucleic acids. By comparing with samples that can be clinically proven, we proposed another molecular diagnostic method of virus diagnosis. The ability to detect nucleic acids in respirator filters is worth investigating in the future, and a study confirmed that they contain a sufficient viral load to enable positive detection of SARS-CoV-2 in the results of dispersal experiments of coronavirus on respirator filters^20^. This was somewhat consistent with our results. Therefore, direct collection and detection of viruses from respirators is highly recommended^12^.

This study was a preliminary study using clinical samples, and there were some limitations. First, regarding the sample size, we acknowledge that the sample size was relatively small. This limitation was primarily due to the study being conducted in a specialized hospital with negative pressure systems, where we focused on patients diagnosed at external COVID-19 screening centers. This limited pool of clinical patients contributed to the constrained sample size. Moreover, another significant limitation was the consideration of potential infection risk and transmission during specimen collection and preparation, particularly in accessing the specialized ward areas. To ensure participant safety, the university hospital's Institutional Review Board (IRB) recommended scaling back large-scale clinical trials, leading us to work within these limitations. Consequently, we were able to include a total of 50 clinical samples in our study after excluding any non-qualified samples. Importantly, this sample size aligns with the predetermined number of samples approved by the IRB. Second, this study did not evaluate the filtration efficiency of masks as other studies have assessed. While we acknowledge the significance of such evaluations, we would like to clarify that our research primarily focused on assessing the diagnostic performance of facemasks rather than evaluating mask filtration efficiency. However, it is important to note that in our original draft, we explicitly mentioned the use of KF94 (or N95)-certified respirators, which have undergone rigorous validation of their filtration efficiency. Moreover, recent studies have indicated that N95-certified masks demonstrate a filtration efficiency ranging from 95 to 99.2%^7,26^. Based on this report, we reaffirm that the majority of exhaled viruses would be captured by the N95-certified masks we employed in our study.

Furthermore, the evaluation of virus particle extraction efficiency from the mask is a crucial aspect of our study. In Supplementary Information, we extensively described and compared two methods (Figs. S1 and S2) for extracting viral RNA from respirators. In the method outlined in Fig. S1, we utilized a 10 mL syringe to contain the mask filter layer with Trizol, enabling the recovery of coronavirus particles from the masks. However, we further enhanced the recovery of virus particles in Fig. S2a–d by adopting a specially designed syringe-centrifuge tube. This new protocol exhibited a moderate increase in liquid recovery, as depicted in Fig. S2e. Importantly, it demonstrated a significant improvement in viral RNA yield, as shown in Fig. S2f. We believe that the observed improvement in viral RNA yield can be attributed to the dominating surface tension within the mask filter membrane pores, a phenomenon that we previously elucidated in our research^27^.

More importantly, RCA tests can provide false positives; therefore, more specific target binding must be developed for an accurate diagnosis. Improvement plans and verification experiments are currently in progress, and we will publish the results soon to suggest a stable alternative. In addition, sensitivity and specificity are the critical criteria in molecular diagnostics. Although research is still in its infancy, this innovative approach represents a refreshing turning point and provides new insights for the future.

Through the present study, we could identify several potential future directions for respiratory virus collection and detection. One crucial area of focus is the optimization and standardization of mask extraction protocols to enhance virus particle recovery efficiency. This would involve exploring innovative approaches and technologies to improve the capture and recovery of viral RNA, such as the utilization of novel materials for mask filters. One promising avenue is the use of water-soluble polymer membranes, which could facilitate the extraction of virus particles from mask filters in a quick and easy manner. In addition, the integration of innovative wearable sensors or immediate detection apparatus holds great potential. For example, the RCA-flow test, as an example of integrating the RCA method with advanced microfluidics, could be further enhanced by incorporating electric biosensors. This integration would enable real-time and on-site detection directly within a facepiece respirator^12,13^. The required liquid for the detection process could be obtained from exhaled breath moisture by utilizing superabsorbent polymers.

## Methods and materials

All procedures performed in this study, including human participants, were in compliance with institutional and/or national research council ethical standards and those of the 1964 Declaration of Helsinki and any subsequent revisions or similar ethical standards. The study protocol was approved by the Institutional Review Boards of Korea University Guro Hospital (Approval No.: 2021GR0092) and Konyang University Hospital (Approval No.: KYUH 2020-12-018-003). The data used in this study are described in Supplementary Information. All experiments were performed in five replicates (n = 5) in each condition throughout the study.

### Respirator collection and storage

Facial respirators were collected from patients with SARS-CoV-2 infection identified by official PCR tests at the COVID-19 Lifestyle Treatment Center operated by Korea University Kuro Hospital and Kongyang University Hospital. Written Informed consent was obtained from all the patients, the patients were requested to wear respirators for up to 4 h. Clinical samples for the experiment were obtained from hospitalized patients (n = 72, male 40, age = 68.7 ± 12.7 yrs), including individuals confirmed positive for SARS-CoV-2 (n = 50), as well as negative controls (n = 22), as outlined in Table 1. The test concordance between the PCR test and the RCA-flow test was evaluated using the same mask samples, as presented in Table 2. However, due to the increase number tests (PCR vs. RCA-flow) for the three viral target genes (R-, N-, RdRp-genes) and the limited availability of extracted samples, the total number of positive samples was reduced to 26. Thus, the samples used in Table 2 were a subset of the samples used in Table 1. Additionally, mask samples were successfully collected from 19 out of the 50 confirmed SARS-CoV-2 cases for three consecutive days (n = 19), as depicted in Fig. 3. All respirators were KF94 certified and approved by the US Food and Drug Administration (FDA) as well as Korea FDA. These respirators were carefully collected in sterilized plastic bags and immediately stored in a freezer at − 80 °C. Samples that did not satisfy the exclusion criteria were excluded from this study. The exclusion criteria for sample collection and storage are as follows: (1) If the respirator was not worn by a confirmed COVID-19 patient; (2) The respirator was worn for an insufficient time (t > 4 h); (3) The storage temperature (< 80 °C) was not met after sample collection.

### Experimental set-up

Figure 1b shows the process of extracting viral RNA from a respirator. To identify SARS-CoV-2, we adopted four target genes: the N, E, RdRp, and ORF1ab genes (Fig. 1c). These target genes are used in many commercially available assays. For example, commercial PCR reagents were purchased from Seegen. The reagents were for the N, E, and RdRp genes as target genes. As shown in Fig. 1d, we adopted two additional tests based on RCA-FL and RCA-flow tests. The RCA-FL test is a common RCA test with fluorescence detection using a PCR device, and the RCA-flow is a rapid test using DNA hydrogel formation in microfluidic pores^19^. KF94 is at least as good as N95 in blocking SARS-CoV-2 particles, as the number 94 indicates the filtration efficiency. Before examining the respirators of patients, we examined the effect of respirator-wearing time on the quantity of nucleic acids collected from the respirators of healthy controls. Note that exhaled breath includes various substances such as DNA, RNA, exosomes, and various volatile organic compounds^28^.

### Cell-free DNA from respirators

Cell-free DNA (cfDNA) is a subset of circulating extra-cellular DNA in plasma, which is released from cells mostly through apoptosis, necrosis, and active secretion. cfDNA has a unique potential as a biomarker for cancer patients or in the field of prenatal care. The extracted cfDNA was quantified using TapeStation system methods. Recent studies have detected circulating cfDNA in the plasma of patients with various types of cancers. The size distribution of cfDNA was mostly similar in the plasma from cancer patients and healthy donors, with an average size of 150–200 bp^20^. The TapeStation system is an established automated electrophoresis tool for DNA and RNA sample quality control, and it can perform unattended analysis of size, concentration, and integrity through fully automated sample processing. It provides a complete solution for true end-to-end sample quality control within any next-generation sequencing (NGS) or Biobank workflow and is well-suited for quality control of DNA samples derived from clinical data.

### Extraction of viral RNA prep from respirators

The process of extracting RNA from the respirator is as follows. The outer portion of the collected mask was trimmed to extract the internal electret filter, which was subsequently cut into dimensions of 50 mm × 50 mm. Then, the cut respirator was placed in 5 mL Trizol, incubate at room temperature (24 °C) for 10 min and rotate. Subsequently, the entire solution was drained from the respirator using an ultracentrifuge. Subsequently, dispense 200 µL of chloroform into 1.5 mL tubes. Add 1 mL of the mixture extracted from the respirator. The mixture was stirred for 15 s and incubated at room temperature for 5 min. After centrifugation at 12,000×*g* and 4 °C for 10 min, only the clear supernatant was isolated. Mix 500 µL of isopropanol with the supernatant, stir the mixture, and incubate at room temperature for 10 min. Centrifuge the samples again at 12,000×*g* and 4 °C for 10 min, then remove the supernatant. The remaining process was the same as the conventional one. After adding 1 mL of 75% ethanol to the RNA from which the supernatant was removed, the mixture was stirred, and centrifugation was performed at 7500×*g*, 4 °C for 5 min, and then the supernatant (ethanol) was removed, and washed twice depending on the sample. Next, the lid of the mixture was opened and dried for 5–10 min (to remove ethanol completely). Finally, after dispensing and pipetting 15 µL of RNase-free water (DW), the mixture was incubated at 65 °C on a heating block for 10 min, followed by incubation on ice for 2 min to extract RNA. After the RNA was extracted from the virus collection respirator, it was quantified with nano-drops, and fragment size distribution was evaluated with a 4200 TapeStation Instrument (Agilent Technologies, Santa Clara, CA, USA).

### PCR test with respirator samples

In this study, we purchased a commercial SARS-CoV-2 detection kit (Allplex™ SARS-CoV-2 Assay, Seegene, Seoul, Korea) that has been FDA-approved as a COVID-19 diagnostic reagent Box for emergency use. This product is a real-time gene amplification (RT-PCR) COVID-19 diagnostic kit that detects three target genes (E, RdRp, N) to confirm SARS-CoV-2 infection. In South Korea, it has been approved for emergency use by the Ministry of Food and Drug Safety. Allele-specific amplification was performed using a real-time PCR system (CFX96 Touch™ Real-Time PCR, Bio-Rad) as follows: First, a reaction master mix was prepared. The master mix contains 5-μL of SARS2 MOM, 5-μL of EM85, and 5-μL of RNase-free water for a total volume of 15 μL of master mix. The total amount of each reagent required is calculated based on the number of reactions, including samples and controls. Vortex master mix and centrifuge briefly; add 15 μL of reaction stock to 0.1 mL 8-tube tubes (MicroAmp™ Fast 8-Tube Strip, 0.1 mL, Applied Biosystems™); add 5 μL of nucleic acid from each sample into the tube containing the reaction master mix. Close the 8-strip cap (MicroAmp™ Optical 8-Tube Cap Strip, Applied Biosystems™) and centrifuge the PCR tube briefly. The liquid containing all PCR components is located at the bottom of each PCR tube.

The thermal profile was set up as follows: 20 min cycles at 50 °C, 15 min cycles at 95 °C, then 45 cycles at 95 °C for 10 s, 60 °C for 15 s, and finally 72 °C for 10 s. Data were analyzed using Bio-Rad CFX Maestro software with cycle threshold (Ct) set to 200.

### RCA-flow test

**Figure 4:**
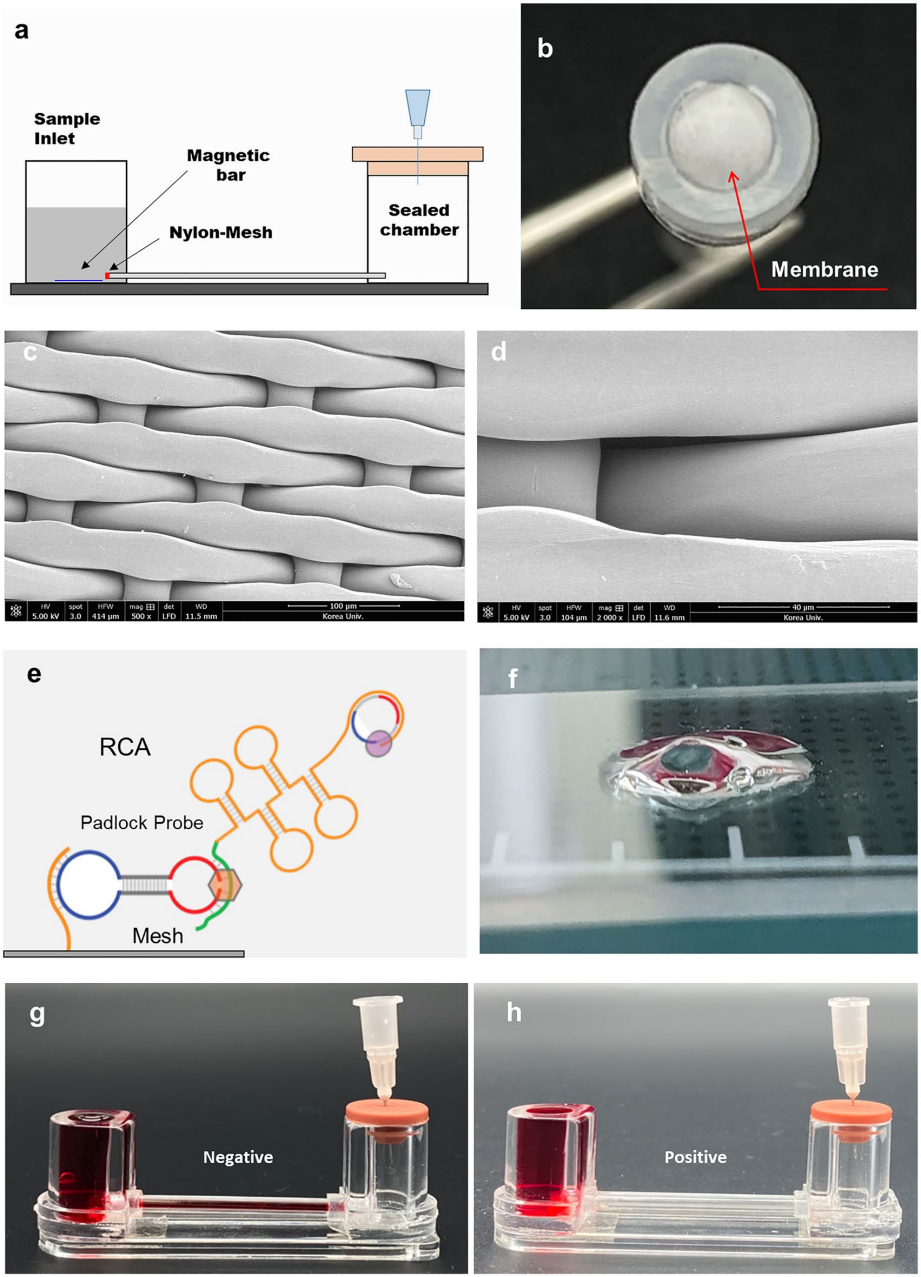
(**a**) Schematic of RCA-flow with microfluidics; (**b**) detailed photograph of a test tube coupled with a nylon mesh to the virus detection unit of the microfluidic chip; (**c**) and (**d**) are high-magnification SEM photographs of the nylon mesh having microscale pores; (**e**) schematics of RCA on nylon mesh surface; (**f**) photo of a hydrogel formed through RCA reaction with RNA obtained from a clinical mask sample; (**g**) negative control for the SAR-CoV-2 pathogen using a microfluidic chip; (**h**) positive control for SAR-CoV-2 confirmed patients using microfluidic chip.

An integrated microfluidic system and its detection results are shown in Fig. 4. The microfluidic system can operate alone without any equipment such as a syringe pump or connecting tubing. Since the liquid level in the sample chamber is higher than the liquid level in the other chamber, the flow is driven by gravity by puncturing the rubber cover. However, when a target pathogen, such as SARS-CoV-2, is present in the sample, a hydrogel is produced that plugs the micropores in the grid^29^. Due to the small size of the micropores in the nylon mesh (about 1 µm), these micropores are rapidly blocked by DNA entanglement through the RCA process. Figure 4c and d show SEM images of nylon mesh at high magnification, while Fig. 4f shows a photo of the hydrogel formed by RCA reaction with RNA obtained from clinical respirator samples. Notably, in the absence of pathogens, there was natural flow owing to gravity, and repeated tests indicated a very good coefficient of variation (< 5%) for migration times.

### Diagnostic performance of respirators with RCA-flow

To confirm SARS-CoV-2 detection using clinical samples, we compared the molecular diagnostic results with the RCA method. Tubes with primer-linked nylon mesh attached were each inserted into the microfluidic kit. This can be confirmed by the information in our previous study^14^. Additionally, a nucleic acid sample from a respirator of a normal person was called a negative control. The flow path through the normal respirator (negative control) was not completely blocked at a specific incubation time of 30 min by individually placing a specific COVID-19 pathogen into the inlet of both microfluidic kits to achieve a fast flow (~ 17 s) through the test tube (Fig. 4g). However, the flow path through which the clinical sample of a confirmed corona patient passed through the primer-bonded nylon mesh was completely blocked and no flow occurred all the way through the test tube (Fig. 4h).

For the RCA flow test, the microfluidic system developed in our recent study was employed^20,21^. The system consists of a sample chamber, glass tube, padlock probe conjugated nylon mesh, and a waste chamber with a rubber cover. Since the waste chamber is sealed with a rubber cap, the sample loaded into the sample chamber cannot flow through the tube. When the rubber cap is connected to the atmosphere, the sample fluid is driven into the waste chamber owing to the increased hydrostatic pressure in the sample chamber. In this RCA flow-test, we employ a very thin grid on which fixed padlock probes capture the target gene. Hybridization occurs when the probe encounters the target pathogen. The opened padlock probe is ligated using ligase (T4 ligase, 12.5 μM) to form a closed-loop template, which can then proceed to the RCA process. Over time, in the RCA process, complementary single-stranded DNA is elongated into a dumbbell shape using Bst 3.0 DNA polymerase. Because of the dumbbell-shaped template, amplified long DNA tends to easily entangle, aggregate with adjacent DNA, and form DNA gels. As a result, the hydrogel partially or completely plugs the micropores of the mesh; thus, depending on the degree to which the hydrogel plugs the micropores, a flow delay or no flow occurs. Detailed information is available in our previous papers^20,21^.

### Supplementary Information

Supplementary Information.

## Data Availability

All data generated or analysed during this study are included in this published article and its supplementary information files.
