# Supplementary material for: Diagnostic performance of respirators for collection and detection of SARS-CoV-2
Source: Sci Rep. 2023 Aug 15;13:13277. doi: 10.1038/s41598-023-39789-w (PMC10427661; doi:10.1038/s41598-023-39789-w)
Supplement: Supplementary file 1 — Supplementary Information. [file 41598_2023_39789_MOESM1_ESM.pdf]

Supplementary Information

Diagnostic performance of respirators for collection and detection of SARS-CoV-2

Hwang-soo Kim[1¶], Hansol Lee[2¶], Seonghui Kang[3], Woo Joo Kim[4*], Sehyun Shin1,[5*]

[1] Department of Micro-nano System Engineering, Korea University, Seoul 02841, Republic of Korea
[2] Asia Pacific Influenza Institute, Korea University College of Medicine, Seoul 02841, Republic of Korea

[3] Department of Mechanical Engineering, Sejong University, Seoul 05006, Republic of Korea

[4] Division of Infectious Diseases, Department of Internal Medicine, Konyang University Hospital, Daejeon 35365, Republic of Korea

[5] Division of Infectious Diseases, Department of Internal Medicine, Korea University College of Medicine, Seoul 02841, Republic of Korea
[6] School of Mechanical Engineering, Korea University, Seoul 02841, Republic of Korea

*Corresponding author:
Sehyun Shin, Ph.D.
Professor
School of Mechanical Engineering, Korea University
Seoul 02841, Republic of Korea
Tel.: +82 2 3290 3377; Fax: +82 2 928 5825
E-mail: lexerdshin@korea.ac.kr

*Co-corresponding author:
Woo Joo Kim, MD, Ph.D.
Professor
Division of Infectious Diseases, Department of Internal Medicine, Korea University College of Medicine
Seoul 02841, Republic of Korea
Tel.: +82 2 2626 3051; Fax: +82 2 2626 1105
E-mail: wjkim@korea.ac.kr

¶ These authors contributed equally to this work.

Fig. S1 Process of isolating and extracting the virus-collected layer from the face mask. (a) Process of separating the filter layer from the face mask. (b) Schematic of the membrane layer of a face mask. (c) Overall process of RNA extraction from the collected mask.

Fig. S2 Method of increasing the amount of recovery of viral nucleic acids from a filter layer. (a)–(d) Protocol for syringe-centrifuge for maximum recovery of the lysis solution from the filter layer. (e) Comparison of recovery of the lysis solution. (f) Comparison of Ct values by quantifying nucleic acids targeting exosome genes using RT-qPCR.

Fig. S3 SARS-CoV-2 detection using DNA hydrogel formation. (a) COVID-19 non-pathogen (negative control), (b) N gene, (c) E gene, and (d) RdRp gene.

Fig. S4 Quantitative trend analysis using SARS-CoV-2 target gene according to the mask collection date. (a) E gene, (b) RdRp gene, and (c) N gene.

Fig. S5 Quantitative trend analysis using SARS-CoV-2 target gene according to each sample type. (a) E gene, (b) RdRp gene, and (c) N gene.

Table S1 List of SARS-CoV-2 template, pathogen, linker primer, and primer nucleic acid sequences used in this study

Table S2 Diagnostic criteria by flow in RCA-flow system.

Materials

Pathogen nucleic acid sequences (26 nt) for COVID-19 (E, N, RdRp, ORF1ab gene); template DNAs (102 nt) for COVID-19 (E, N, RdRp, ORF1ab gene); primers (NH_2-polyA9-primer (NH2 primer: 32 nt), and additional primers (12 nt) were synthesized by Bioneer (Daejeon, Korea) (Table S1). Rigid Teflon tubes (ID = 0.4 mm, OD = 0.9 mm) and flexible silicon tubes (ID = 0.8 mm, OD = 1.8 mm) were purchased from Sungjin Rubber Co. (Seoul, Korea). Nylon mesh with a 1-μm pore was purchased from ELKO Filtering Co. (Florida, USA). Bst 3.0 DNA polymerase, 10× Isothermal Amplification Buffer II, T7 ligase, and pyrophosphatase were obtained from New England Biolabs (Ipswich, MA, USA). A total of 25 mM dNTP was obtained from Thermo Fisher Scientific Inc. (MA, USA) and 100 mM DTT was purchased from Epi-center Technologies Corp. (Madison, USA). Bovine serum albumin (BSA) and adenosine triphosphate (ATP) were obtained from Sigma-Aldrich (St. Louis, MO, USA). The amine coupling kit (A515-10: NHS buffer, WSC buffer, activation buffer, reaction buffer, and blocking buffer) was purchased from Dojindo (Kumamoto, Japan). A T100 thermal cycler was purchased from Bio-Rad (Hercules, CA, USA). Other chemicals were purchased from Sigma-Aldrich (St. Louis, MO, USA).

Immobilization of Linker Primer on Nylon Mesh Surface

After the washing step, 200 μL of 100-mM NHS buffer and 200 μL of 100-mM WSC buffer (both from the coupling kit) were mixed with nylon mesh and incubated at room temperature (~23 °C) for 30 min. The nylon mesh was then washed twice with 500 μL of 1×PBS buffer. The washed nylon mesh was then resuspended in 380 μL of reaction buffer and 20 μL of 1-mM NH2 primer and incubated for 2 h. The nylon mesh was then washed twice with 500 μL of 1×PBS buffer, resuspended in 500 μL of blocking buffer and incubated for 1 h. The nylon mesh was washed twice and resuspended in 400 μL of 1×PBS buffer.

Extraction of SARS-CoV-2 virus nucleic acid from face mask

The RNA extraction process from the mask involved the following steps. Initially, the facemask was carefully opened, and the filter membrane was extracted. Subsequently, the membrane was cut into a size of 50 mm x 50 mm and placed inside a 10 mL syringe for sample extraction. 5 mL of Trizol was then dispensed into the syringe, ensuring that it covered the membrane of the mask. The syringe with the Trizol-covered membrane was incubated at room temperature for 10 minutes. After incubation, the entire solution was withdrawn from the syringe and divided into 1 mL tubes each. Then, 200 µl of chloroform was dispensed into the 1 ml solution. It was then shaken for 15 seconds and incubated for 5 minutes at room temperature. After centrifugation at 12,000 g, 4° C., for 10 minutes, only the transparent supernatant was separated. After mixing 500 µl of isopropanol with the supernatant, we incubated it for 10 min at room temperature after stirring. It was centrifuged again at 12,000 g, 4 °C, 10 min, and then the supernatant was removed. After adding 1 ml of 75% ethanol to the RNA from which the supernatant was removed, we it centrifuged at 7500 g, 4 °C, 5 min, and then removed the supernatant (ethanol). It was dried (to remove ethanol completely) for 5–-10 min with the lid open, and washing was performed twice depending on the sample. Finally, after dispensing 15 µl of RNase-free water (DW) and pipetting, we incubate it at 65 °C for 10 min in a heating block, and then incubate it on ice for 2 min to extract RNA.

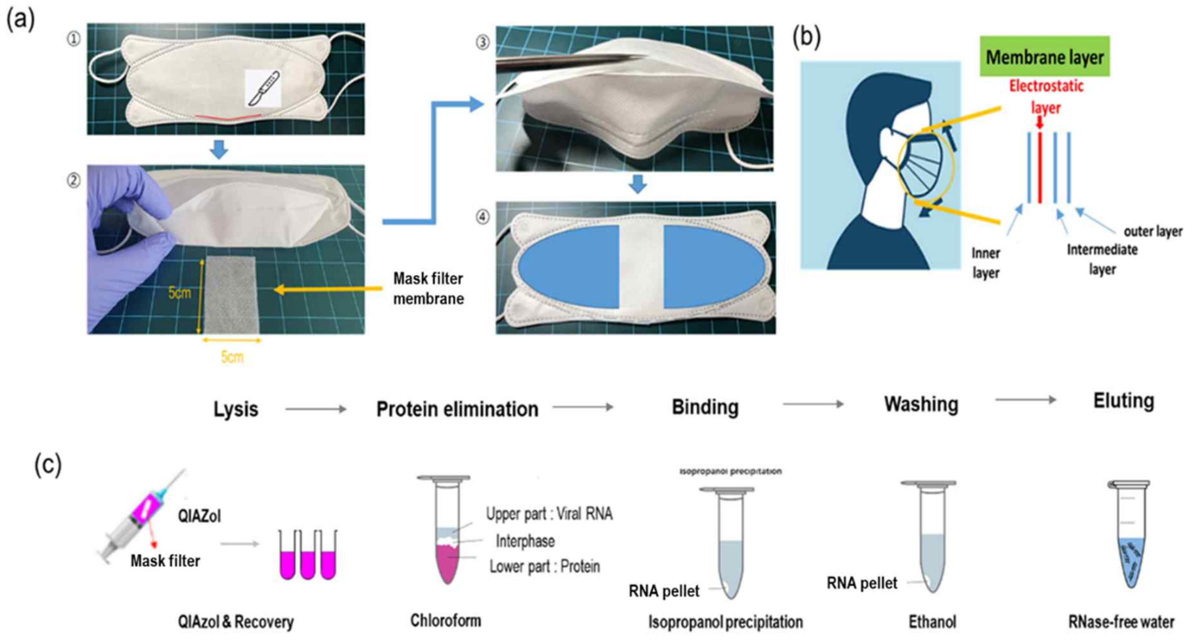

Fig. S1 Process of isolating and extracting the virus-collected layer from the face mask. (a) Process of separating the filter layer from the face mask. (b) Schematic of the membrane layer of a face mask; the face mask consists of four layers (outer, intermediate, filter, and inner layers). (c) Overall process of RNA extraction from the collected mask.

Increase in the amount of recovery of viral nucleic acids from the filter layer

To enhance the recovery of viral nucleic acids from the filter layers, we utilized a centrifuge with a syringe. Initially, the mask membrane was cut to the appropriate size (as depicted in Fig. S1(a)-S1(d)). Subsequently, the mask membrane was incubated in 5 ml of Trizol at room temperature for 10 minutes and then placed inside the body of a 10 ml syringe. The syringe body, containing the mask membrane, was inserted into a 50 ml tube. Using a centrifuge set at 7000 g for 15 minutes, the entire solution was extracted from the mask.

When employing 5 ml of lysis buffer within the mask, approximately 4-4.4 ml (80%-86%) of the solution could be recovered using a syringe. However, by utilizing the centrifuge method, over 4.8 ml (95%) of the solution could be successfully recovered. The extraction protocol plays a crucial role in increasing the concentration of viral nucleic acids and addressing the concentration-related issues. Higher recovery rates of the mask lysis solution, exceeding 95%, can potentially lead to an increase in concentration. As demonstrated in Fig. S2(e), we evaluated the recovery volume of the lysis solution using both the syringe and centrifuge methods. The centrifugation technique yielded a relatively higher recovery volume. Furthermore, as shown in Fig. S2(f), we were able to quantify the nucleic acids targeting viral genes through RT-qPCR and compare their Ct values using the centrifuged samples, which exhibited relatively higher concentrations.

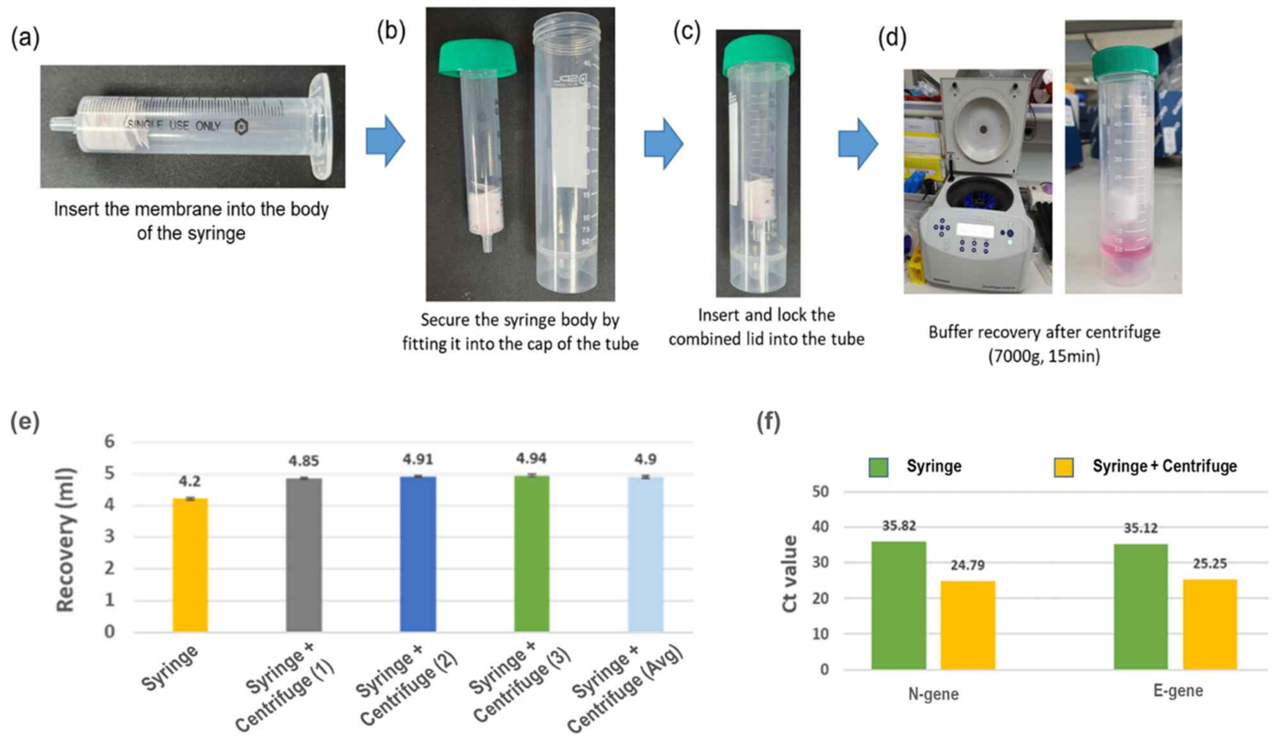

Fig. S2 Method of increasing the amount of recovery of viral nucleic acids from a filter layer. (a)–(d) Protocol for syringe-centrifuge for maximum recovery of the lysis solution from the filter layer. (e) Comparison of recovery of the lysis solution. (f) Comparison of Ct values by target genes using RT-qPCR.

Validation of DNA hydrogel formation by SARS-CoV-2 templates

To prepare the RCA mixture, we obtained 1 μL of 100 μM clinical mask sample (containing RNA), 1 μL of T4 ligase (400 U/μL), 1 μL of pyrophosphatase (100 U/mL), 4 μL of phi29 DNA polymerase (10 U/μL), and 40 μL of a 10x phi29 reaction buffer. The 10x phi29 reaction buffer consisted of 4 μL of polymerase buffer, 1 μL of 100 mM DTT, 4 μL of 25 mM dNTP, 1 μL of 50 mM ATP, 1 μL of 100 μM additional primer, 1 μL of BSA (10 mg/mL), and 21 μL of distilled water. To confirm the formation of DNA hydrogels, we added various types of SARS-CoV-2 templates to the RCA mixture and incubated it at 30 °C for 60 minutes. The dumbbell-shaped templates have a tendency to promote entanglement and aggregation with neighboring DNAs, resulting in the formation of DNA hydrogels.After incubation, we observed the formation of significant DNA hydrogels in the tubes containing the N, E, and RdRp gene templates of SARS-CoV-2. In contrast, no DNA hydrogel formation was observed in the tube without the SARS-CoV-2 template. The captured image (Fig. S3) clearly demonstrates the strong formation of DNA hydrogels depending on the presence of the target template.

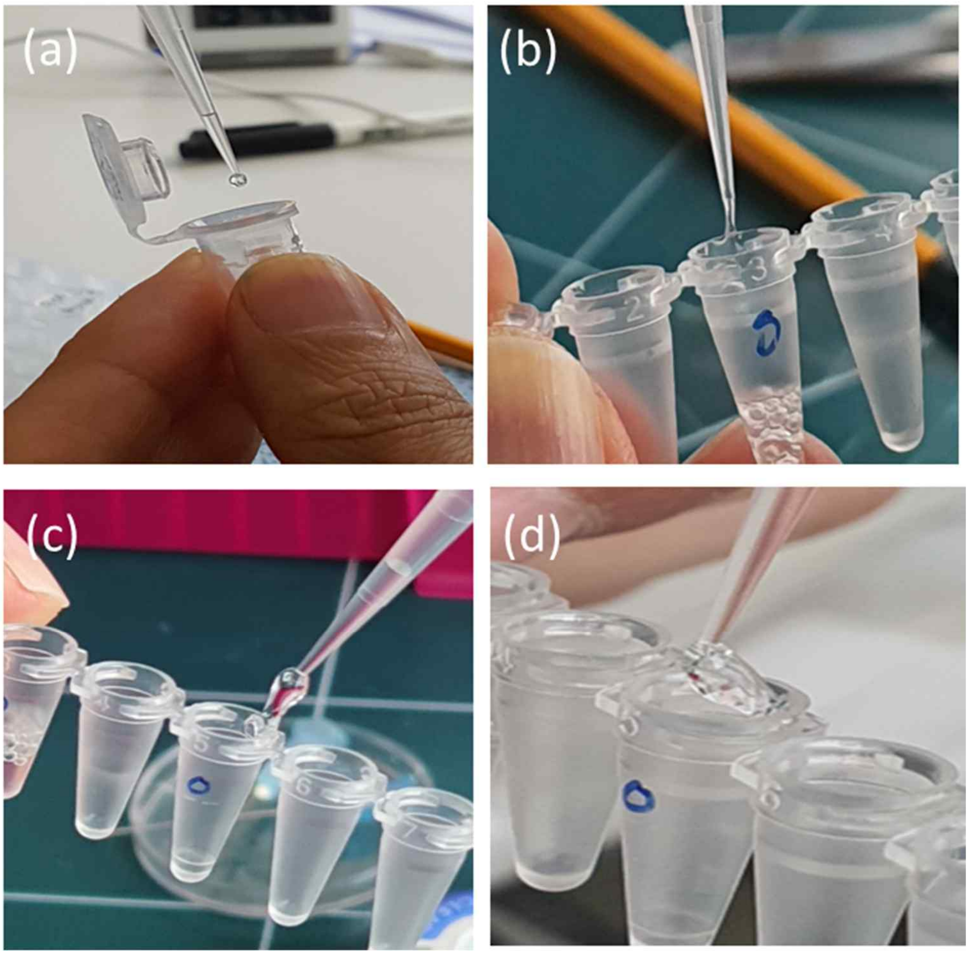

Fig. S3 SARS-CoV-2 Detection using DNA hydrogel formation. (a) COVID-19 non-pathogen (negative control), (b) N gene, (c) E gene, and (d) RdRp gene.

Comparison of quantitative values for each SARS-CoV-2 target gene according to the date of mask collection

Analysis was conducted using a total of N=9 masks collected over a span of three days. Quantification was performed through RT-PCR, targeting the SARS-CoV-2 genes (E, RdRp, and N genes). Fig. S4 (a) to (c) presents the results, indicating that the overall Ct value for each target gene exhibited a trend of shifting towards higher values over time. This observation suggests that the viral load tended to decrease as the days of collection progressed.

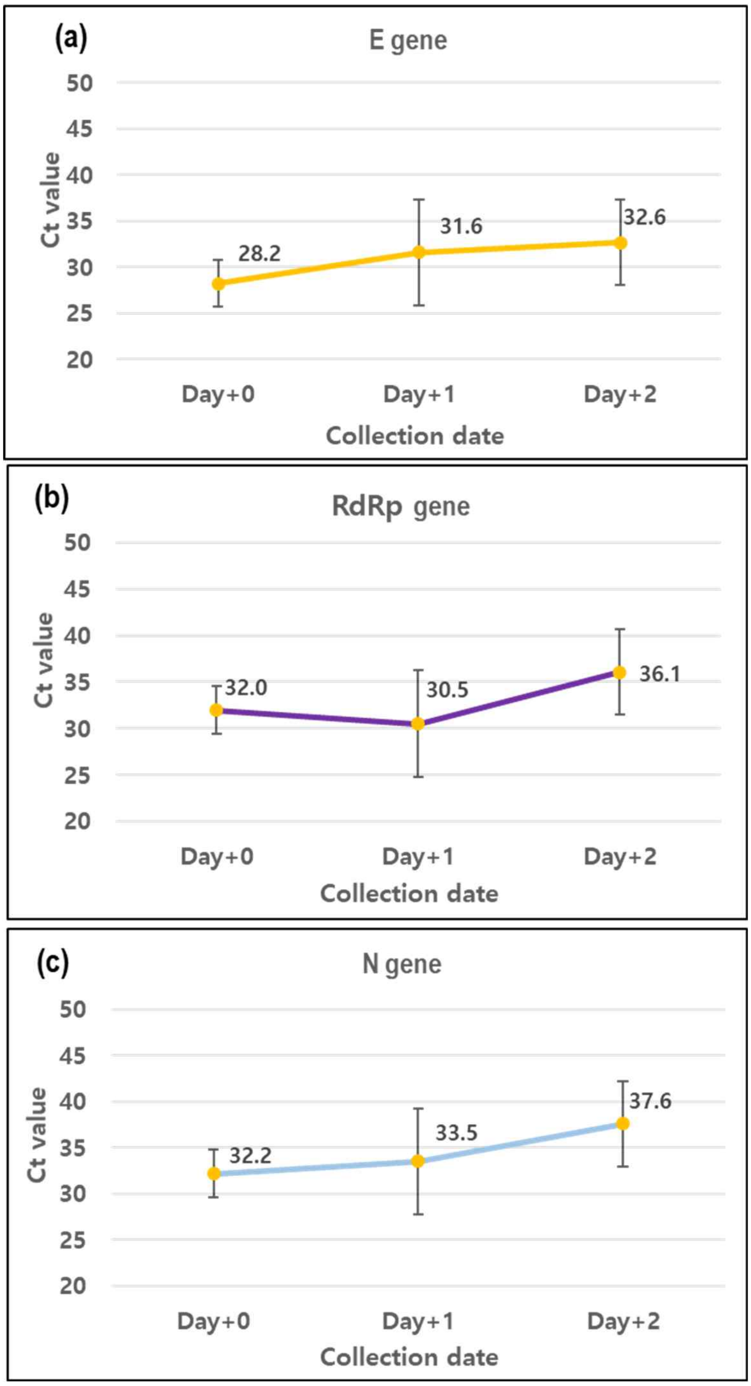

Fig. S4 Quantitative trend analysis by SARS-CoV-2 target gene according to the mask collection date. (a) E gene, (b) RdRp gene, and (c) N gene.

Comparison of quantitative values for SARS-CoV-2 target gene according to each sample

Analysis was conducted on each sample type (nasal swab, saliva, mask) with N=3 samples for each type. Virus quantification was carried out using RT-PCR, employing the target genes of SARS-CoV-2 (E, RdRp, N genes).

Fig. S5 (a) to (c) illustrates the results obtained, indicating that there was no significant variation in the Ct value across the different target genes based on the sample type. This suggests that the efficacy of nucleic acid collection from the mask is comparable to that of conventional sample collection methods. Therefore, our findings demonstrate that the performance of mask-based sample collection is not significantly inferior to traditional approaches.

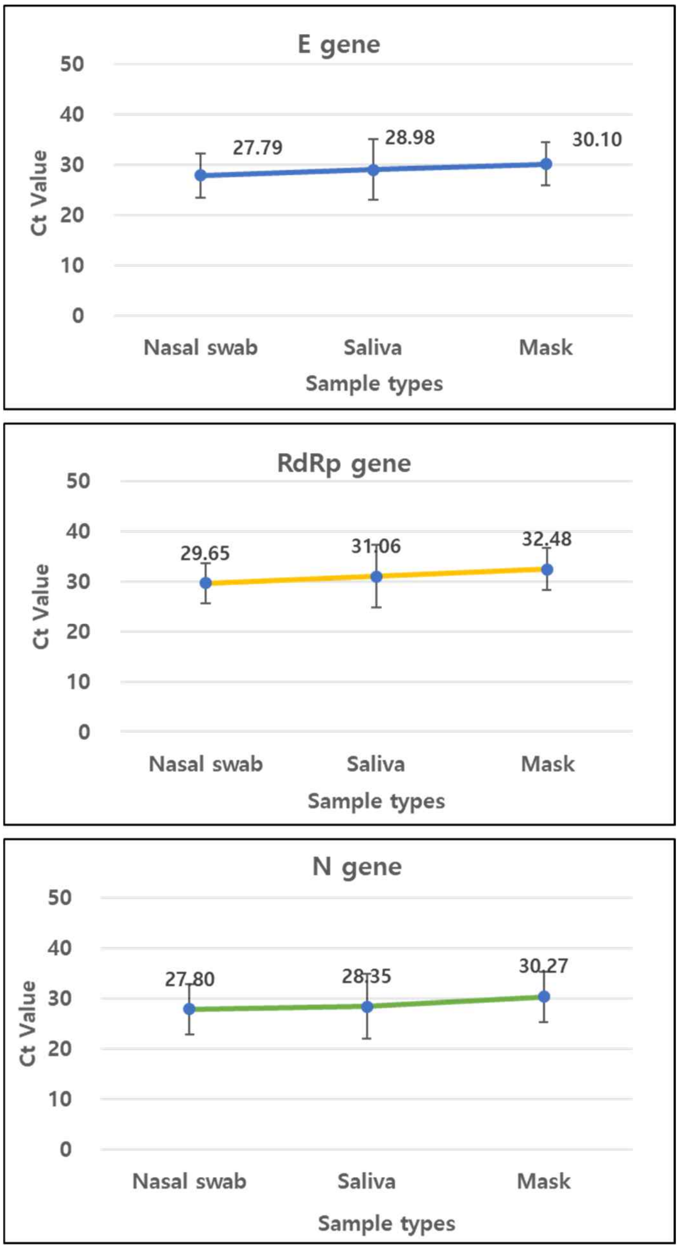

Fig. S5 Quantitative trend analysis by SARS-CoV-2 target gene according to each sample type. (a) E gene, (b) RdRp gene, and (c) N gene.

Table S1. List of SARS-CoV-2 template, pathogen, linker primer, and primer nucleic acid sequences used in this study.

Strands	Sequence (5' → 3')
COVID-19 N gene template (102 nt)	5'-phosphate-AAT ACC ATC TT A ATC GAA GTA CTC AGC GTA AGT TTA GAG GTA GCA TGC TAG TAT CGA CGT CCC ACG TAC CAA CTT ACG CTG AGT ACT TCG ATT GGT AGT AGA-3'
COVID-19 E gene template (102 nt)	5'-phosphate-CAC GTT AAC AAA ATC GAA GTA CTC AGC GTA AGT TTA GAG GTA GCA TGC TAG TAT CGA CGT CCC ACG TAC CAA CTT ACG CTG AGT ACT TCG ATT TAC AAG ACT-3'
COVID-19 RdRp gene template (102 nt)	5'-phosphate-GAA CTT CCT TCA ATC GAA GTA CTC AGC GTA AGT TTA GAG GTA GCA TGC TAG TAT CGA CGT CCC ACG TAC CAA CTT ACG CTG AGT ACT TCG ATT AAT TCA ACA-3'
COVID-19 ORF1ab gene template (102 nt)	5'-phosphate-GTA GCC ATA CTA ATC GAA GTA CTC AGC GTA AGT TTA GAG GTA GCA TGC TAG TAT CGA CGT CCC ACG TAC CAA CTT ACG CTG AGT ACT TCG ATT AAG TAG TAT-3'
COVID-19 pathogen(26 nt)_ N gene	5'- TAA AAG ATG GTA TTT CTA CTA CCT TA-3'
COVID-19 pathogen(26 nt)_ E gene	5'- TAA TTG TTA ACG TGA GTC TTG TAT TA-3'
COVID-19 pathogen(26 nt)_ RdRp gene	5'- TAA GAA GGA AGT TCT GTT GAA TTT TA-3'
COVID-19 pathogen ORF1ab (26 nt)	5'- TAA AGT ATG GCT ACA TAC TAC TT TTA-3'
NH2-polyA-primer (32 nt)	5'-Amino(C6)-AAA AAA AAA GGG ACG TCG ATA CTA GCA TGC TA-3'
Additional Primer (12 nt)	5'-TGC TAG TAT CGA-3'

*Red indicates the pathogen binding site, green indicates the self-assembly region, and blue indicates the primer binding site.

Table S2. Diagnostic criteria by flow in RCA-flow system.

<table>
<tr><th>Flow</th><th>Result</th></tr>
<tr><td>Block</td><td rowspan="2">Detected(+)</td></tr>
<tr><td>Touch down
≥ 20 s</td></tr>
<tr><td>Touch down
≤ 20 s</td><td>Not detected(-)</td></tr>
</table>
